# Dissecting Regulators of Aging and Age-Related Macular Degeneration in the Retinal Pigment Epithelium

**DOI:** 10.1155/2022/6009787

**Published:** 2022-11-16

**Authors:** Pabalu P. Karunadharma, Rebecca J. Kapphahn, Madilyn R. Stahl, Timothy W. Olsen, Deborah A. Ferrington

**Affiliations:** ^1^Department of Ophthalmology and Visual Neurosciences, University of Minnesota Twin Cities, MN 55455, USA; ^2^Graduate Program in Biochemistry, Molecular Biology, And Biophysics, University of Minnesota Twin Cities, Minneapolis, MN 55455, USA

## Abstract

Age-related macular degeneration (AMD), the leading cause of blindness in elderly populations, involves the loss of central vision due to progressive dysfunction of the retinal pigment epithelium (RPE) and subsequent loss of light-sensing photoreceptors. While age is a key risk factor, not every aged individual develops AMD. Thus, the critical question is what specific cellular changes tip the balance from healthy aging to disease. To distinguish between changes associated with aging and AMD, we compared the RPE proteome in human eye bank tissue from nondiseased donors during aging (*n* = 50, 29-91 years) and in donors with AMD (*n* = 36) compared to age-matched donors without disease (*n* = 28). Proteins from RPE cells were separated on two-dimensional gels, analyzed for content, and identified using mass spectrometry. A total of 58 proteins displayed significantly altered content with either aging or AMD. Proteins involved in metabolism, protein turnover, stress response, and cell death were altered with both aging and AMD. However, the direction of change was predominantly opposite. With aging, we detected an overall decrease in metabolism and reductions in stress-associated proteins, proteases, and chaperones. With AMD, we observed upregulation of metabolic proteins involved in glycolysis, TCA, and fatty acid metabolism, with a concurrent decline in oxidative phosphorylation, suggesting a reprogramming of energy utilization. Additionally, we detected upregulation of proteins involved in the stress response and protein turnover. Predicted upstream regulators also showed divergent results, with inhibition of inflammation and immune response with aging and activation of these processes with AMD. Our results support the idea that AMD is not simply advanced aging but rather the culmination of perturbed protein homeostasis, defective bioenergetics, and increased oxidative stress within the aging RPE, exacerbated by environmental factors and the genetic background of an individual.

## 1. Introduction

Age-related macular degeneration (AMD) is the leading cause of blindness in individuals over the age of 65 in developed countries and globally the third leading cause of vision loss [1, 2]. This disease manifests as a loss of central, high-acuity vision, caused by the death of the retinal pigment epithelium (RPE) and photoreceptors in the macular region of the retina. Patients with advanced AMD have difficulties performing essential daily functions, such as reading, writing, and driving, thereby significantly impacting their quality of life [2]. While the exact cause of AMD is still not completely resolved, most of the experimental evidence points to the dysfunction and death of the RPE as a critical pathogenic event. Located between the retinal photoreceptors and the outer retinal blood supply of the choroid, the RPE performs many key functions that helps maintain vision. These functions include daily phagocytosis of the oldest portion of photoreceptor outer segments, transport of nutrients and oxygen from the choroid to the outer retina, and secretion of molecules that are crucial for the health and integrity of the retina and choroid [3].

Multiple environmental (e.g., high-fat diet and smoking) and genetic risk factors (e.g., single nucleotide polymorphisms in complement genes) have been linked to an increased likelihood for developing AMD. However, age remains the strongest risk factor as approximately 30% of individuals over 75 years develop AMD [4]. Since not every aged individual develops AMD, the critical question is what specific cellular changes tip the balance from healthy aging to disease. Distinguishing molecular changes that occur during aging from changes with AMD onset and progression will provide important new insight into how the RPE responds to the challenges of aging and disease.

Currently, there are no reliable animal models that fully replicate the key characteristics of AMD due to the involvement of the macula, which is unique to primate eyes, and the age-dependent onset of the disease. Our laboratory investigates the biochemical changes occurring in the retina using human donor eyes that have been characterized for the presence and severity of AMD by the Minnesota Grading System (MGS) [5]. The MGS incorporates clinical phenotypes defined by the Age-Related Eye Disease Study (AREDS), considered to be a standard in clinical trials, to classify human donor eyes [6]. Thus, the biochemical phenotypes observed at each stage in donor retinas are directly relevant to a patient's clinical phenotype.

In two prior studies, we employed a proteomics approach to begin defining how retinal proteins are altered with AMD progression. A global proteome analysis of the RPE found content of 12 proteins localized to the mitochondria were altered with AMD [7]. This initial study provided the first indication that defects in RPE mitochondria may play a role in AMD pathology. Subsequent studies of the RPE mitochondria from AMD donor tissue have shown mtDNA damage and dysfunction of the energetics [8, 9]. The current study extends our previous work by including an analysis of RPE protein changes during aging (*n* = 50, 29-91 years) performed in parallel with a comparison of donors at progressive stages of AMD (*n* = 36) compared with their age-matched nondiseased controls (*n* = 28). Based on a review of studies applying proteomics to analyze RPE [10], the current study provides the most comprehensive examination of human RPE proteome since it spans a significant range in ages and stages of AMD to distinguish how aging and disease affect the RPE.

## 2. Materials and Methods

### 2.1. Human Tissue Procurement

Donor eyes were obtained from the Lions Gift of Sight (formerly Minnesota Lions Eye Bank, St. Paul, MN, USA) with written consent either from the donor or the donor's family for use in medical research in accordance with the principles outlined in the Declaration of Helsinki. Criteria established by the Minnesota Grading System (MGS) were used to determine the donor's disease stage from high resolution photomicrographs [5]. MGS 1 represents the control group with no AMD. MGS 2, 3, and 4 are early, intermediate, and advanced stages of AMD, respectively. Donor demographics included time and cause of death, a limited medical history and ocular history (Table S1). RPE was dissected fresh and frozen at -80°C until analysis. Exclusion criteria for the present study include a history of diabetes or glaucoma, clinical symptoms of diabetic retinopathy, advanced glaucoma, and myopic degeneration or atypical debris in the eyes.

### 2.2. Protein Isolation

RPE cells used in this study were dissected fresh by gently separating from the Bruch's membrane in a balanced saline solution. It was pelleted at 1100 × *g* and frozen at −80°C until processing. Cells from the peripheral RPE were processed as described [11]. Briefly, pelleted RPE cells from a pair of globes were homogenized in a buffer containing 20 mM HEPES, 10 mM KCl, 1.5 mM MgCl_2_, 250 mM sucrose, 1 mM EDTA, 1 mM EGTA, 1 mM phenylmethylsulfonyl fluoride, and 0.5% NP40. The cells were subjected to two freeze-thaw cycles and gently passed six times through a 26-gauge needle. The lysate was cleared of nuclei, intact cells, and plasma membrane fragments by centrifugation at 600 × *g* for 15 minutes at 4°C. The supernatant was retained, and the pellet was subjected to a second mechanical homogenization and centrifugation as described above. The second supernatant was combined with the first and centrifuged at 13,000 × *g* for 15 minutes at 4°C to pellet the mitochondria. The resultant supernatant enriched for cytoplasmic proteins was used in this study. Protein concentrations were determined using the bicinchoninic acid protein assay (Pierce Biotechnology, Rockford, IL, USA), with bovine serum albumin as the protein standard.

### 2.3. 2D Gel Electrophoresis

The conditions for strip rehydration, focusing, equilibration, and 2D gel electrophoresis were performed as outlined [11]. RPE protein (125 *μ*g) were dissolved in a rehydration solution (9 M urea, 3 M thiourea, 6% CHAPS, 1% ASB-14, 1% Biolytes pH 3–10 (Bio-Rad), and 50 mM dithiothreitol) and incubated with 11 cm IPG strips (pH 5 to 8 linear gradient) (Bio-Rad, Hercules, CA, USA). Proteins separated in the first dimension were resolved on 12% polyacrylamide gels. The 2D gels were stained with Flamingo™ Fluorescent Gel Stain according to the manufacturer's protocol. Gels for mass spectrometry were stained with silver using a mass spectrometry-compatible kit (Silver Stain Plus Kit; Bio-Rad).

### 2.4. Two-Dimensional (2D) Gel Spot Quantification and Analysis

Flamingo-stained gels were imaged using a Dark Reader Transilluminator (Clare Chemical Research, Dolores, CO, USA) and a ChemiDoc XRS (Bio-Rad) imager system. Gel images chosen for analysis were based on the saturation of a standard protein run in a separate lane in the gel. Spot alignment and density quantification were performed using a 2D gel analysis software (PDQuest 7.1.1; Bio-Rad). Prior to spot matching, artefactual noise was removed from each 2D gel using the filter options in PDQuest. Automatic spot detection and matching were followed by manual inspection and editing. Spot intensities of each gel were normalized to the total intensity of valid spots of that gel. Natural log transformed spot density values were used for statistical analysis.

### 2.5. In-Gel Digestion and MS Analysis

Spots were manually excised from 2D silver-stained gels and in-gel digestion performed with trypsin as described [11]. After trypsin digestion, extracted peptides were purified using “stage tips” containing a plug of “Empore” disk (3M, Minneapolis, MN, USA) [12]. LC-MS/MS analysis of trypsin digested 2D PAGE protein spots was carried out using an LTQ Orbitrap mass spectrometer (Thermo Fisher Scientific, San Jose, CA) equipped with a Flex nano-ESI source (Thermo Fisher Scientific, San Jose, CA). Data dependent scanning was performed using Xcalibur v 2.1.0 software, a survey mass scan at 60,000 resolution in the Orbitrap analyzer scanning mass/charge (m/z) 360-1800, followed by collision-induced dissociation (CID) tandem mass spectrometry (MS/MS) of the 5 most intense ions in the linear ion trap analyzer [13]. Precursor ions were selected by the monoisotopic precursor selection (MIPS) setting with selection or rejection of ions held to a ±10 ppm window. Dynamic exclusion was set to place any selected m/z on an exclusion list for 20 seconds after a single MS/MS. Tandem mass spectra were searched against a human FASTA protein database downloaded from UniprotKB on March 09, 2017, which at that time contained 20121 protein entries. All MS/MS spectra were searched using Thermo Proteome Discoverer 1.4 (Thermo Fisher Scientific) considering fully tryptic peptides with up to 2 missed cleavage sites. SEQUEST (embedded with Proteome Discoverer) was searched with a fragment ion mass tolerance of 0.80 Da and a parent ion tolerance of 10.0 PPM. Carbamidomethyl of cysteine (57.021 Da) was specified in SEQUEST as a fixed modification. Deamidation (0.984 Da) of asparagine and glutamine and oxidation (15.995 Da) of methionine were specified in SEQUEST as variable modifications. Protein and peptide identification results were visualized with Scaffold v 4.11 (Proteome Software Inc., Portland OR), a program that relies on various search engine results (*i.e*.: SEQUEST, X!Tandem, MASCOT) and which uses Bayesian statistics to reliably identify more spectra [14]. Accepted proteins passed a minimum of 3 peptides identified at a 95% protein and peptide confidence by the Peptide and Protein Prophet algorithm, within Scaffold [15].

### 2.6. Statistical Analysis

To recognize significant linear changes in protein spot density with aging, we performed regression analysis using GraphPad Prism (version 9). Significant spot quantity changes in the diseased donors (AMD) compared to age-matched controls were examined using two models. Linear density changes across disease stages were tested with linear regression and stage-specific changes using one-way ANOVA and Dunnett's post hoc test for means comparison. The results are expressed as mean ± SEM (AMD data). Statistical significance was set at *p* ≤ 0.05 for both aging and AMD comparisons.

### 2.7. Bioinformatic Analysis

Categorization of proteins into class, molecular function, and biological process was done according to Gene Ontology notation using the PANTHER (version 16.0) classification system [16]. The Kyoto Encyclopedia of Genes and Genomes (KEGG) mapper tool was utilized to categorize the proteins into functional categories [17]. Pathway analysis was performed using the Ingenuity Pathway Analysis (IPA, Qiagen Inc.). Significantly changing proteins from 2D spots that had 3 or fewer protein identifications were utilized for pathway and gene ontology analysis. For the aging IPA analysis, the spot densities were converted to a ratio for comparing pathway changes across different age groups. Donors were separated into 4 age groups: group 1 (29-48 yrs), group 2 (53-63 yrs), group 3 (65-76 yrs), and group 4 (79-91 yrs). The spot densities for each age group were averaged and normalized to group 1. This ratio was input into IPA with *p* values for expression analysis. AMD protein changes were submitted as an expression ratio by normalizing averaged protein density values at each stage to MGS 1 (control) along with *p* values.

## 3. Results

### 3.1. Donor Characteristics

**Figure 1:**
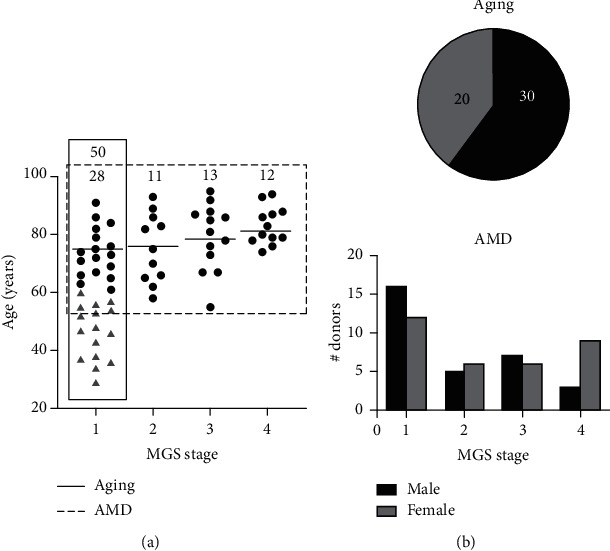
Donor ages and gender distribution in the aging and AMD comparison. (a) Age of donors used in this study. Solid line (—) outlines the control donors used in the aging comparison (*n* = 50). Triangles are donors aged 29–60 (*n* = 22). Circles are donors aged 61–91 (*n* = 28). These donors are also the age-matched controls for AMD comparison. Dashed line (---) outlines the age-matched donors used in the AMD comparison (*n* = 64). Number of donors for each MGS stage is shown at top. Mean age of each age-matched MGS group indicated by line. There was a significant increase in age in MGS 4 compared to controls (one-way ANOVA, *p* = 0.0502). (b) Pie chart showing gender distribution in aging study. Bar graph showing gender distribution per MGS stage.

A summary of the donor demographics and clinical information (provided from Eye Bank records) is presented in Figure 1 and Table S1. All donor eyes were evaluated for the presence and severity of AMD using the Minnesota Grading System [5]. Control donors without AMD (MGS 1) ranging in age from 29 to 91 years old (*n* = 50) were used to determine protein changes that occur with normal aging (Figure 1(a), solid box). Twenty-eight of the MGS1 donors (ages 61-91) were the age-matched controls for determining protein changes that occur at early (MGS 2, *n* = 11), intermediate (MGS 3, *n* = 13), and late (MGS 4, *n* = 12) stages of AMD (Figure 1(a), hatched box). Donors in the MGS 1 group consisted of 20 females and 30 males (Figure 1(b)). There was a relatively balanced distribution of each sex in the AMD analysis except for MGS4, consisting of 9 females and 3 males.

### 3.2. Proteomic Analysis of RPE with Aging and AMD

**Figure 2:**
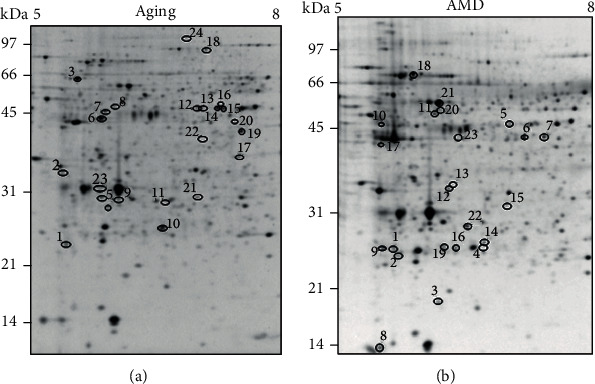
RPE proteins resolved by 2D gel electrophoresis. Representative flamingo-stained gels (125 *μ*g) from the (a) aging and the (b) AMD comparison. Spots indicated showed altered content with each process. A linear range of pI 5 to 8 is indicated at the top and molecular weights of the standard are marked on the left.

**Figure 3:**
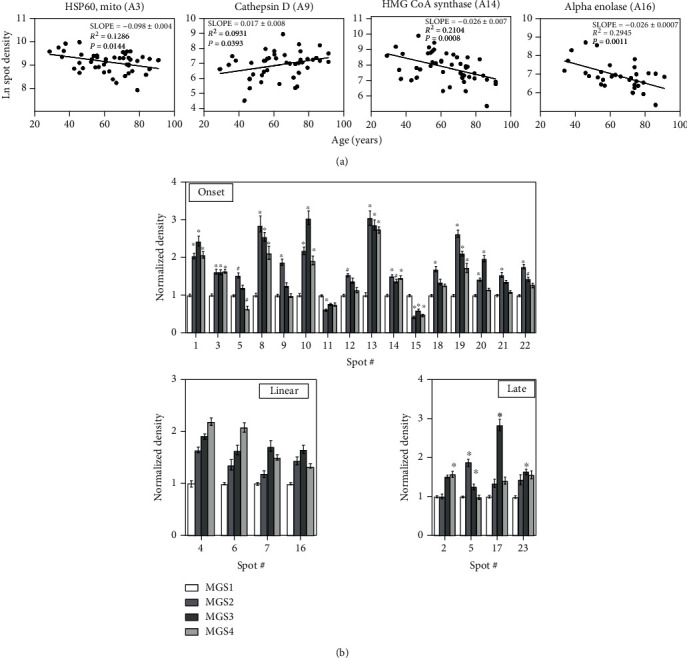
Spots altered with aging and AMD. (a) Four representative spots significantly changing with age. Age in years in the *x*-axis and Log_e_ transformed spot densities are on the *y*-axis. Slope, coefficient of correlation, and regression *p* value are shown for each spot. Additional spots from the aging analysis are listed in Table 1 and Table S2. (b) Average densities at each MGS stage for spots demonstrating significant changes at disease onset, linear over disease progression, and at late stage are shown. Data are the means (±SEM) normalized to MGS 1. Asymmetric error bars: back transformed density values from Log_e_. One-way AOVA with Dunnett's post hoc test was used to determine if different than MGS1. ^∗^*p* < 0.05. *n* = 6 or more per group. All proteins identified from the AMD analysis are listed in Table 2 and Table S3.

Average protein yield for the isolated RPE protein fractions was 534 ± 36 *μ*g (mean ± SEM). There was no significant difference in protein yield among MGS stages (*p* = 0.2, *n* = 64). Proteins were separated by 2D gel electrophoresis and stained with Flamingo Fluorescent Gel Stain prior to analysis of spot density (representative Flamingo-stained gels are shown in Figure 2). For the aging comparison (MGS 1 only), 465 spots were resolved and analyzed (Figure 2(a)). A total of 519 spots were analyzed in AMD age-matched donors (Figure 2(b)). For clarity, we are using “A” for gel spots from the aging analysis and “D” for gel spots from the AMD disease analysis. In the aging comparison, twenty-four spots showed significant linear density changes (Figures 2(a) and 3(a) show a subset, Table 1, and Table S2). Five spots increased in spot density with age, while 19 spots demonstrated a decrease (Table 1 and Table S2).

For the comparison between MGS stages, 23 spots changed with disease progression (Table 2, Table S3). Three distinct patterns of spot density change were observed, including (1) at disease onset (starting at MGS 2), (2) linear changes with disease progression, and (3) at later stages (MGS3 or MGS4, Figure 3(b)). Sixteen spots changed significantly at disease onset (MGS 2), 4 exhibited a linear response, and 4 others were altered at later stages of AMD (Figure 3(b)).

### 3.3. Protein Identification

Proteins were identified by LTQ-Orbitrap mass spectrometry. A high confidence match to a specific protein was based on the criteria of at least 3 unique peptides matching with greater than 95% protein and peptide probability [15]. Seventy-eight unique proteins were identified from all the significantly altered gel spots from both analyses (Tables 1, 2, S2 and S3). Approximately 50% of the significantly changing spots in the aging and AMD comparisons matched a single protein. On average, 26% of the spots in both analyses matched 2 unique proteins and 8% matched 3 proteins. Tables 1 and 2 (also Tables S2 and S3) provide a list of proteins identified in each spot, along with information about significance, molecular weight, sequence coverage, and number of unique peptides identified for each protein. Approximately 20% of the 2D gel spots identified 4 or more proteins (Table S2, S3). These proteins were not considered in subsequent pathway analysis.

The comigration of multiple proteins in a single 2D gel spot or a single protein migrating at different isoelectric points (pI) reflects challenges associated with 2D gels when resolving a highly complex mixture of proteins. Posttranslational modifications (PTM), such as phosphorylation, acetylation, or oxidation, can alter the intrinsic charge of a portion of the protein population by introducing a charge or quenching an existing one, thereby making single proteins migrate at different pIs. An example among the proteins in the aging comparison is HMG CoA Synthase (HMGCS2, 52 kDa, pI 8.16) and alpha Enolase (ENO1, 47 kDa, pI 7.39), which were identified comigrating in four spots (A12-15) at ~48 kDa with an isoelectric point (pI) of 7.1 to 7.4. HMGCS2 was previously observed to show an acidic shift in the pI and attributed to oxidatively modified thiols [18]. ENO1 was observed to migrate in a “charge train” previously, and multiple PTMs were identified including phosphorylated Serine, acetylation at Lys, and methylation at several sites [19]. Importantly, all comigrating spots exhibited a decrease with aging, thereby providing greater confidence in our conclusions for these proteins.

Proteolytic processing from the inactive to active form or removal of a signal sequence can also result in identification of an individual protein in multiple spots that are migrating at different apparent molecular masses. Cathepsin D provides an example where it is synthesized as a single chain polypeptide (pre-pro-enzyme 48 kDa) in the endoplasmic reticulum and transported to the lysosomal compartment via the trans-Golgi network for maturation [20]. The pro-Cathepsin D undergoes proteolytic processing that ultimately produces the mature 37 kDa protein, which exists as both a single polypeptide or can be further processed to form a two-chain polypeptide consisting of the 27 kDa heavy and 10 kDa light chain [21]. We identified Cathepsin D in multiple spots in the aging analysis, migrating at 33 kDa (A2) and ~27 kDa (A4, A9) that demonstrated divergent changes with aging. This result suggests age-related changes in the proteolytic processing of Cathepsin D.

### 3.4. Summary of Functional Categories

To provide a more comprehensive analysis of how aging or AMD affects the RPE proteome, we utilized multiple pathway analysis software to assist in summarizing our findings. We included proteins from gel spots that had 3 or fewer proteins identified by mass spectrometry. For the AMD analysis, we also included results from our previous studies that investigated global protein changes in RPE homogenates and in an enriched RPE mitochondrial fraction (Table S4) [7, 8, 22]. Our rationale for including these data was based on the similarity of protocols in each study, including tissue handling and processing by the Lions Gift of Sight Eye Bank and using the Minnesota Grading System for evaluating AMD severity for individual donors. Using results from all our studies would also increase our ability to map changes associated with AMD to specific biological pathways. Of the 25 proteins identified previously [7, 8, 22], three mitochondrial-localized proteins (Elongation factor Tu, GST-*π*, and HSPA9) were also identified in the current study. The overlap of altered proteins in different donor populations provides greater confidence in the relevance of these findings to AMD.

**Figure 4:**
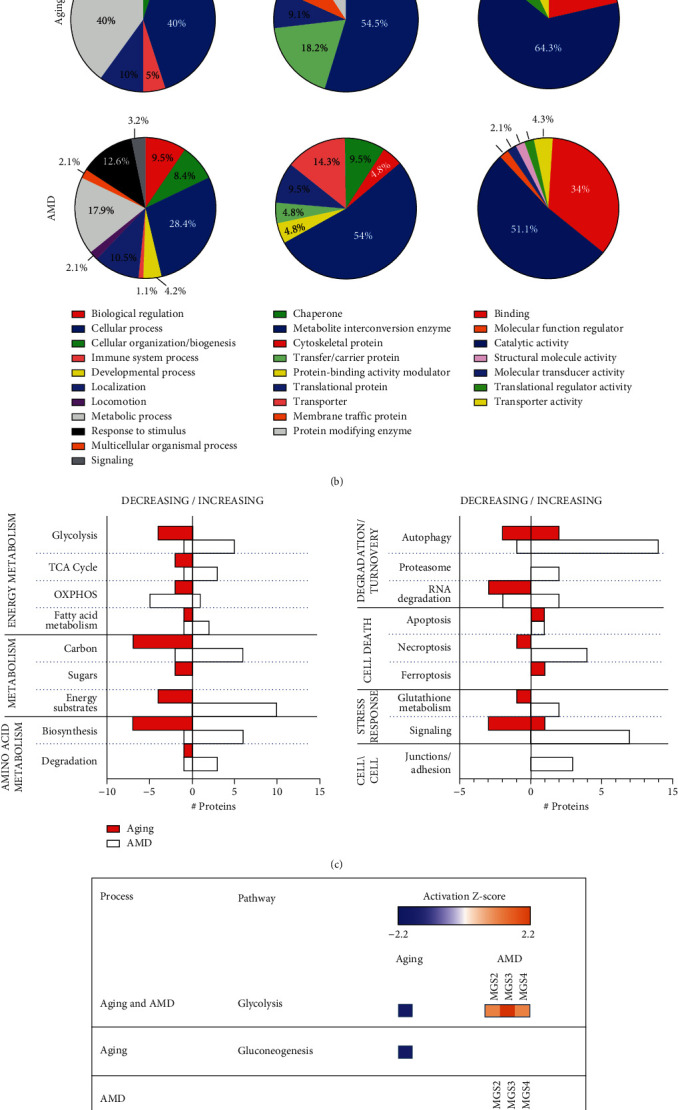
Comparison of functional categories represented in the aging and AMD proteomic analyses. (a) Venn diagram comparing proteins associated with altered 2D spot quantities in the aging and AMD analyses. A total of 15 proteins were identified from the aging analysis and 35 proteins from the AMD comparison. Only 8 proteins were common to both processes. (b) Functional classification of differentially expressed proteins in aging and AMD. Pie charts demonstrate the distribution of proteins according to their protein class, molecular function, and biological function based on Gene Ontology notation and PANTHER online tool for categorization. (c) Comparison of proteins identified from the aging and AMD analyses graphed based on direction of change. Proteins categorized to functional groups based on KEGG mapper were plotted based on whether they increased or decreased with aging or AMD. *y*-axis shows the functional categories, and *x*-axis the number of proteins increasing or decreasing. (d) Canonical pathways with significant activation Z-scores (-2.23 ≥ *Z* <1.5) are shown for aging and AMD after Ingenuity pathway analysis. Blue color depicts inhibition and orange activation.

Eight identified proteins were common to both aging and AMD, while 15 and 35 proteins were unique to aging and AMD, respectively (Figure 4(a) Venn). ENO1 (glycolysis), HMGCS2 (mitochondrial ketogenesis), and HSP60 (mitochondrial chaperone) were three proteins that decreased in both aging and AMD. The remaining five proteins, which decreased with aging and increased with AMD, were from metabolic pathways (PGAM11, GLUL, and IDH1), mitochondrial protein synthesis (TUFM), and from the visual cycle (RLBP1). The preponderance of metabolic proteins changing with both aging and AMD provides an early indication that altered energy production and utilization may be one of the key processes affected under both conditions.

Protein identifications were mapped to biological process, protein class, and molecular function, using the Panther classification system (Figure 4(b)) [16]. In considering each classification, proteins from AMD donors showed a more diverse distribution involving a greater number of categories. These results suggest that AMD has a more complex and broad effect on the proteome. For both aging and AMD, the predominant protein class was metabolic enzyme, which is consistent with major changes in energy utilization for both aging and AMD.

To further distinguish the protein changes in top functional categories, we analyzed our data using the KEGG mapper classification system [17]. The number of proteins associated with each KEGG pathway was plotted with their direction of change (Figure 4(c)). Major categories involving metabolism, protein turnover, stress response, and cell death were impacted heavily with both aging and AMD, but notably, the direction of change was predominantly opposite between the two processes. For example, proteins in the metabolism, degradative pathways, and stress response were decreased with aging and increased with AMD. Specific to AMD was the upregulation of three proteins involved in cell junctions and adhesion (INSR, RHOA, and ACTB), potentially reflecting the disease-induced disruption in RPE tight junctions and connection with Bruch's membrane. These results show divergence between pathways associated with retinal aging that is free of AMD and pathways changing with AMD progression.

To gain additional insight into the top altered pathways, downstream effects, and upstream regulators, the differentially expressed proteins were analyzed by the Ingenuity Pathway Analysis (IPA, Qiagen Inc.). The top pathways in the aging and AMD analyses that significantly changed (Fisher's exact test ≤0.05) with an absolute Z-score between -2.23 ≥ *z* <1.5 are shown in Figure 4(d). Glycolysis was significantly altered in both groups, although opposing patterns of activation or inhibition was observed (Figure 4(c)). With aging, glycolysis and gluconeogenesis were predicted to be significantly inhibited (Z-score ≥ -2), while AMD showed activation from MGS2 (Figure 4(d)). This suggests a strong impact in this quintessential pathway with both aging and disease in the RPE. HIF1 alpha signaling pathway showing activation with AMD suggests a significant response to oxidative stress with disease. Other pathways that were inhibited with AMD include oxidative phosphorylation (OXPHOS), PPARa/RXRa activation, estrogen receptor signaling, and xenobiotic metabolism. The inhibition of these critical signaling pathways suggest disruption to the nutrient and damage sensing network with AMD.

**Figure 5:**
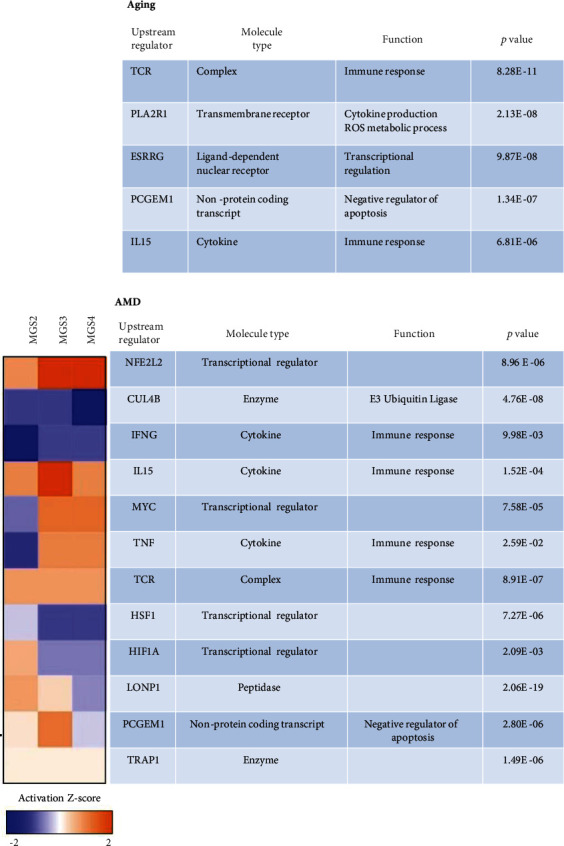
Upstream regulator analysis for aging and AMD. Upstream transcriptional regulators (TRs) predicted by IPA to be significantly altered given the protein changes detected with aging (top) and AMD (bottom). Significance reported using Fisher's exact test *p* values calculated based on the overlap between protein changes and genes regulated by the TR. Activation Z-scores comparing MGS 2, 3, and 4 show inhibition (blue) or activation (orange) when compared with MGS1.

**Figure 6:**
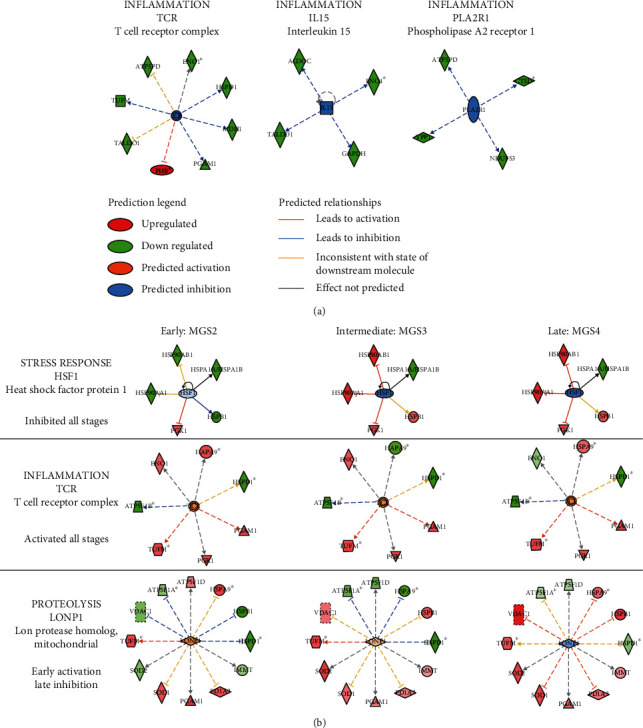
Upstream regulator analysis from IPA. Representative networks showing upstream regulators and target molecules from the data set for the aging (a) and AMD (b) analysis. Upstream regulators and differentially expressed proteins are displayed as nodes and edges (biological relationship between nodes). The color intensity of each node represents expression: red (upregulated), and green (down regulated). The edges connecting the proteins to the proposed upstream regulator represent the predicted relationships: blue inhibition, orange activation, and gray no effect predicted based on the IPA activation Z-score. Additional networks are in Supplemental Figure S1.

The IPA upstream regulator analysis provides insight into the biologically significant changes upstream of the observed protein changes and can identify transcription factors or any other molecules that have been experimentally observed to affect gene/protein expression (Figures 5 and 6). For aging, IPA identified 5 potential regulators (Figure 5). Three of the regulators (TCR, PLA2R1, and IL15) predicted to be inhibitory (Figure 6(a)) are associated with inflammation and immune response. Prostate-specific transcript 1, also known as PCGEM1, is a long noncoding RNA that is hypoxia-responsive and a negative regulator of apoptosis. Estrogen-related receptor gamma (ESRRG), a nuclear receptor, regulates transcription of genes involved in mt biogenesis, OXPHOS, TCA cycle, glycolysis, and fatty-acid oxidation [23]. Both regulators were predicted to be inhibited based on the protein changes associated with aging (Figures 5 and S1A).

AMD upstream analysis revealed 12 regulator molecules predicted to be significantly affected (Figures 5 and 6(b), Figure S1B). Similar to aging, several immune regulators were affected with AMD including TCR (Figure 6(b)), TNF, IL-15, and IFNG (Figure 5, Figure S1B). Notably, all immune regulators were activated with disease progression except IFNG. This agrees with previous findings of increased inflammation and immune response with AMD progression [24]. Additionally, several transcription factors involved in oxidative stress and hypoxia were affected with AMD. MYC and NFE2L2 were predicted to be activated (Figures 5 and S1B). MYC is a master regulator of transcription that control many genes involved in cell division, chromatin modification, ribosome, and mt biogenesis [25]. NFE2L2 is also a major transcription factor that upregulates expression of antioxidant proteins and protects against ROS-induced oxidative damage [26]. Two other transcriptional regulators of the oxidative stress response, HSF1 (Figure 6(b)) and HIF1A (Figure S1B), showed inhibition at later stages of AMD but activation at MGS2 (Figure 5, Z-score heat map). This suggests an early response to oxidative stress and the increased response to hypoxia due to thickening Bruch's membrane and drusen accumulation that starts early in AMD [27–31]. Two other regulators showed a similar pattern of early activation followed by inhibition at later disease. They include PCGEM1, a negative regulator of apoptosis, and LONP1, a mitochondrial protease that functions as a chaperone and assists with mt OXPHOS subunit assembly and mt protein biogenesis [32]. One additional mitochondrial protein tumor necrosis factor receptor-associated protein 1 (TRAP1), also known as heat shock protein 75 (Hsp75), is a mitochondrial member of the Hsp90 family of molecular chaperones [33]. It acts as a key regulator of mitochondrial bioenergetics by downregulating OXPHOS and promoting glycolysis to reduce reactive oxygen species and increase mt tolerance to oxidative stress [34]. Thus, TRAP1 activation predicted with AMD (Figure 5) could be an indication of the metabolic reprograming with AMD and attenuation of mt energetics.

## 4. Discussion

In the current work, a parallel proteomic comparison of aging and AMD was performed using RPE proteins extracted from human donor eyes categorized for the presence and severity of AMD. The use of donor eyes in conjunction with the MGS provided a platform to identify altered retinal proteins and pathways to distinguish normal aging from pathology. Our results show that while there is overlap between aging and AMD, the majority of protein changes were unique to each process (Figure 4(a)). Importantly, the expression profile for most major categories was divergent comparing the two processes; the content of most proteins was decreased with aging and increased with AMD (Figure 4(c)) suggesting that normal aging and AMD are distinct processes.

The process with the most dramatically contrasting proteome when comparing biological aging versus pathology was the proteome changes in metabolic pathways (Figures 4(c) and 4(d)). Proteins from the glycolytic pathway (ALDOC, GAPDH, PGM1, and ENO1), TCA cycle (Transaldolase 1 and MDH1), electron-transport chain (ATP5PD and NDUFS3), and ketogenesis (HMG CoA synthase 2) were decreased with aging (Table 1). IPA pathway analysis suggested a significant decline in both glycolysis and gluconeogenesis, including predicted inhibition of ERR gamma signaling (ESRRG, Figure S1 Aging) that regulate the above-mentioned metabolic pathways. These results, as well as another study [35], are consistent with a general decline in metabolic activity in the aging RPE.

In contrast to aging, increased glycolytic proteins (ENO1, PGM1, PGK1, PKM, and ALDH2) and decreased content of OXPHOS proteins (NDUFV2, PPA2, COX6B1, ATP5F1A, ATPF1B, and ATPF1C) were observed with AMD progression (Figures 4(c) and 4(d), Table S4). These results are consistent with measurements of ATP production in primary RPE cultures from AMD donors where the majority of the ATP was generated via glycolysis and not from OXPHOS [36]. Notably, the significant loss in RPE mitochondrial function in cultures from AMD donors was corroborated in another study [37]. Results from proteomic analysis as well as functional analysis in primary RPE is consistent with the idea that AMD has a significant negative impact on RPE mitochondria. Other data using different experimental approaches also strongly implicate mitochondrial dysfunction as a pathologic feature of AMD. Supportive data from human donor tissue include ultrastructural defects in RPE mitochondria [38], altered protein content in RPE mitochondria and choroid/Bruch's membrane [39], increased mtDNA damage in macular RPE from AMD donors [9, 40], and decline in mt ETC proteins and function observed in a recent large-scale proteomic study on differentiated RPE cells from AMD patients [41]. For example, depletion of mtDNA in the RPE cell line, ARPE-19, using ethidium bromide, resulted in induction of genes indicating a switch in metabolism from oxidative phosphorylation to glycolysis and fatty acid metabolism as a mechanism for maintaining energy production [42]. Results from the current study, including upregulation of proteins in glycolysis, TCA, and fatty acid metabolism, suggest a reprogramming of energy utilization and provide further support for the hypothesis that mitochondrial dysfunction is a pathologic feature of AMD.

While our results suggest significant metabolic restructuring has occurred with AMD in RPE, it is important to note that we cannot directly infer causality as the protein changes simply correlate with disease. However, important insight about how alterations in the RPE mitochondrial function could initiate AMD has been developed using transgenic mice, which show the existence of a highly coordinated “metabolic ecosystem” between photoreceptors and RPE [43–45]. In this model, RPE transports glucose from choroidal blood to the photoreceptors where it is utilized for aerobic glycolysis. The by-product lactate is transported from photoreceptors back to the RPE and used for ATP production via OXPHOS. Data show that under normal conditions, glycolysis is suppressed in the RPE due to the high levels of lactate received from the retina [45]. However, with AMD, the loss in RPE mitochondrial function and subsequent decrease in ATP production would trigger a greater reliance on glycolysis to supply RPE energy demands. The change in energy source to glucose could ultimately starve the underlying photoreceptors of glucose that is required for their energy needs. Decreased photoreceptor glycolysis could eventually lead to degeneration and cell death, a hallmark of AMD.

Another major class of proteins affected with both aging and AMD include chaperones and proteases that participate in the stress response. With aging, proteins involved in degradation (TPP1 and Cathepsin D) and refolding (HSP60, prohibitin, and ERP29) were significantly altered (Figure 4(c)) with most proteins decreasing in content. In our AMD analysis, GRP75 (mtHSP70, #D18), a mitochondrial matrix chaperone, and PDIA3 (#D21) an endoplasmic reticulum (ER) chaperone, were increased at onset (Figure 3(b)). IPA upstream regulator analysis showed activation of TRAP1 (Figure S1B) and activation of LONP1 at MGS2 with decline at later stages (Figure 6(b)). Since these proteins function as mt chaperones that assist with OXPHOS assembly and reduce oxidative stress, these patterns of activation agree with an early increase in oxidative stress in RPE that affect mt protein folding and homeostasis. Previous work from our lab suggest damage to mtDNA and altered content of ETC and mt heat shock proteins (mtHSP70, mtHSP60) from the earliest stage of AMD [7, 8, 22]. Damage to mtDNA and chaperones can consequently lead to production of damaged mtDNA-encoded proteins and improper folding and transport of nuclear-encoded proteins. Proteolysis is also affected, based on the increase in the 20S proteasome observed in RPE with disease progression [22]. The processes of protein refolding and degradation are essential for maintaining proteostasis. Deficiency in proteostasis could be especially detrimental to postmitotic RPE that is involved in many functions to maintain retinal health throughout life. Also, the contrast in the protein alterations between aging and pathology indicate distinct differences between the two processes, suggesting significant effects on AMD pathogenesis by disrupting protein homeostasis.

Several key proteins involved in oxidative stress response were upregulated at AMD onset (GST *π*, SOD1, Peroxiredoxin-3, Aldh2, glutamine synthetase). The observed upregulation of these redox-sensitive proteins may represent a compensatory response to increased oxidative stress in the RPE, which is consistent with a growing body of evidence implicating cumulative oxidative damage in AMD pathogenesis [46]. For example, the aged SOD1-deficient mouse demonstrated drusen-like deposits, choroidal neovascularization, and RPE abnormalities similar to AMD suggesting that oxidative stress is a major event in AMD pathogenesis [47]. We observed a significant increase in SOD1 content from MGS2 onwards (Figure 3(c), spot #D3). GST *π* is an inhibitor of proapoptotic signaling and stabilizes reactive electrophiles in response to increased oxidative stress [48–50]. For mitochondrion-specific Peroxiredoxin-3, considered a first line of defense against superoxide-derivative hydrogen-peroxide in the mitochondria, we observed a steady increase in Peroxiredoxin-3 (>2-fold) with progression of AMD (Figure 3(c), spot #D4) [51]. Mitochondrial ALDH2 (Figure 3(c), spot #D20) is essential for the removal of toxic aldehydes that are by-products of lipid peroxidation in the RPE [52]. In a recent study, overexpression of ALDH2 restored retinal function by reducing apoptosis and enhancing chaperone activity in mice [53]. Glutamine synthetase is an enzyme that generates glutamine via the condensation of glutamate and ammonia. Glutamine is one of the three amino acids that make up the important cellular redox regulator, glutathione [54]. Thus, upregulation of glutamine synthetase with AMD could indicate a requirement for more glutathione in response to increase oxidative stress. Previously enhanced utilization of glutathione was observed in primary RPE cultures when subjected to an oxidative stress [55]. Brown and colleagues showed that elevated oxidative stress is sufficient for RPE loss of function including mt disruptions, photoreceptor structural alterations, and a metabolic shift in the RPE to glycolysis in a RPE-specific SOD2-deficient mouse model [43]. Therefore, results from the current study and our previous studies showing increased content of multiple redox-sensitive proteins with AMD is consistent with increased oxidative stress as a major contributor AMD pathology [22].

In our aging analysis, IPA pathway and upstream regulator analysis predicted a decline in TCR, PLA2R1, and IL15 (Figure 6(a)), and this agrees with reduced T-cell receptor repertoire, lower production of naïve T-cells and interleukin's, and an inhibition of adaptive immunity that is observed with normal aging [56]. But with AMD, predicted activation of the inflammatory markers TCR, TNF, and IL15 (Figure 6(b), S1B) along with the apparent increase in oxidative stress (as discussed above) that is highly intertwined with inflammation agrees with prior reports that associate an inflammatory imbalance with AMD. Also supporting this idea are genetic association studies that linked the complement pathway (CFH, CFB, CFHR1/CFHR3, C3, and C5) to AMD, as well as the presence of complement proteins identified in significant quantity in retinal drusen [57, 58]. AMD inflammatory processes include complement activation, microglia activation, and macrophage infiltration [59]. Therefore, the immune system is a major player in AMD where immune dysregulation in the aging retina could be exacerbated by increased complement activation, increased proinflammatory cytokines, and activation of microglia in response to oxidative stress and soft-drusen accumulation.

In this study, we resolved and quantified ~500 protein spots from a complex mixture of RPE proteins. While this represents only a portion of the total RPE proteome, we identified 58 proteins that exhibited a significant change with either aging or AMD. There are several caveats associated with our experimental design and analysis, including that the proteins identified herein represent a conservative estimate of the total proteins changing due to the limitations inherent in 2D gels. With our sample size, a 50% change in spot density was the lower limit of change required for statistically significance. However, much smaller, physiologically relevant differences may have gone undetected. In the analysis of protein density changes with aging, linear regression analysis was used to identify age-dependent changes and would miss nonlinear changes that are biologically meaningful. In the first dimension of separation, a pH range of 5 to 8 was utilized because it provided the greatest resolution for the majority of spots; however, protein changes outside this range would not be detected. While we were able to identify several plasma membrane proteins (i.e., prohibitin, apolipoprotein A-1, and transforming RhoA) from spots that showed differential content, membrane proteins resolve poorly in the first dimension and may be underrepresented. Given these caveats, we are currently employing proteomics techniques with more sensitivity and quantitative accuracy using state-of-the-art mass spectrometry and label-free proteomic quantification to extend and validate findings from the current study,

## 5. Conclusion

In summary, we used a proteomic approach to compare protein changes associated with normal aging and AMD in human donor RPE. While our results show some overlap in the biological processes affected, such as metabolism, energy utilization, and inflammation, a divergent direction of change was observed. Changes unique to aging include an overall decline in metabolic proteins and a decrease in chaperones and proteases, which refold and degrade damaged proteins to maintain cellular proteostasis. There was also inhibition of inflammation and immune response. The overall suppression of processes that protect against invading pathogens and cellular damage could establish an environment that promotes disease, especially in the presence of environmental or genetic risk factors.

The proteomic profile of AMD was distinct from that observed with aging. For example, in contrast to the decrease in glycolysis observed with aging, glycolytic enzymes were increased with AMD. This metabolic switch that favors glycolysis is likely due to defects in mitochondrial function, as shown by multiple studies [36, 42] and reflected in the observed decreased content of OXPHOS proteins. There was also multiple indicators of elevated oxidative stress and activation of inflammation and the immune response. The important contribution of an overactive inflammatory response in AMD development was recently proposed by two different groups who promoted the idea that the cumulative damage induced by aging, accompanied by the chronic inflammation, sets the stage for AMD pathogenesis [57, 60]. What “tips the balance” to disease is how the host responds to these challenges, which depends on the individual's genetic background. In general, our results are consistent with this idea.

## Figures and Tables

**Table 1:** Proteins identified from spots changing with Aging (3 or less protein ID spots).

Spot no.†	*p* value	*R* ^2^	Direction‡	Protein ID	Accession UniProt	Gene	Experimental MW/pI§	Theoretical MW/pI ¥	MSMS scaffold		
									Sequence coverage	Unique peptides	Total spectra
A1	0.0319	0.09428	D	ATP synthase subunit d, mitochondrial	O75947	ATP5PD	21/5.6	18/5.30	30	4	6
				Ras-related protein Rab-18	Q9NP72	RAB18		23/5.11	17	3	3

A2	0.0148	0.1364	D	Transducin *β*-1	P62873	GNB1	33/5.5	37/6.00	45	9	17
				Retinaldehyde-binding protein 1	P12271	RLBP1		36/5.05	25	7	15
				Cathepsin D	P07339	CTSD		37.9/5.6 €	11	3	3

A3	0.0144	0.1286	D	HSP 60, mitochondrial	P10809	HSPD1	62/5.7	61/5.87	65	34	101

A4	0.0003	0.2497	D	NADH dehydrogenase iron sulfur protein 3, mitochondrial	O75489	NDUFS3	25.5/6.0	30/7.5	19	4	10
				Cathepsin D	P07339	CTSD		26.7/5.56 ₡	11	3	3

A5	0.0075	0.1933	D	Prohibitin	P35232	PHB	27/5.9	30/5.76	46	10	19

A6	0.0057	0.01576	D	Creatine kinase B	P12277	CKB	44/5.9	43/5.89	20	5	6
				Tripeptidyl-peptidase 1	O14773	TPP1		39.8/5.75 ∆	8	3	7

A9	0.0393	0.0931	I	Cathepsin D	P07339	CTSD	27.2/6.2	26.7/5.56 ₡	26	6	19

A12	0.0004	0.2391	D	Alpha enolase	P06733	ENO1	47.5/7.1	47.2/7.01	51	16	60
				HMG CoA synthase, mitochondrial	P54868	HMGCS2		52/8.16 *δ*	18	7	10

A14	0.0008	0.2104	D	HMG CoA synthase, mitochondrial	P54868	HMGCS2	47.5/7.2	52/8.16 *δ*	35	15	26

A15	0.0002	0.2558	D	Alpha enolase	P06733	ENO1	47/7.4	47/7.39	44	15	47
				HMG CoA synthase, mitochondrial	P54868	HMGCS2		52/8.16 *δ*	17	6	7

A16	0.0011	0.2945	D	Alpha enolase	P06733	ENO1	52/7.3	47/7.39	44	15	47

A17	0.0027	0.2233	D	Aldose reductase	P15121	AKR1B1	35/7.5	36/6.98	38	9	16
				Malate dehydrogenase, cytoplasmic	P40925	MDH1		36/7.39	21	5	5
				GAPDH	P04406	GAPDH		36/8.46	18	4	4

A18	0.0274	0.1082	I	Serotransferrin	P02787	TF	86/7.1	77/7.12	60	36	107

A19	0.0346	0.0955	D	Fructose-bisphosphate aldolase C	P09972	ALDOC	43/7.5	39/6.87	67	17	89

A20	0.0229	0.1228	D	Glutamine synthetase	P15104	GLUL	44.5/7.4	42/6.89	26	9	14
				Isocitrate dehydrogenase [NADP] cytoplasmic	O75874	IDH1		47/7.01	25	9	14
				Elongation factor Tu, mitochondrial	P49411	TUFM		50/7.61	13	4	4

A21	0.0314	0.3558	D	Phosphoglycerate mutase 1	P18669	PGAM1	29.6/7.1	29/7.18	19	5	11

A22	0.02	0.149	D	Transaldolase	P37837	TALDO1	42.5/7.1	38/6.81	19	6	6

A23	0.061 ^∗∗^	0.2286	I	Prohibitin	P35232	PHB	30.5/5.9	30/5.76	58	12	23

A24	0.0079	0.2124	I	Programmed cell death 6-interacting protein	Q8WUM4	PDCD6IP	97/6.9	96/6.52	13	9	10

† Spot number indicated on gel picture in Figure 2 left panel. Only spots with ≤3 proteins identifications are listed in this table. The remaining spots are listed in Table S2. ‡ Direction increasing (I) or decreasing (D) with age. § Experimental molecular weight (MW) of each spot was calculated based on the relative mobility of the MW markers and distance traveled on the 2D gel. Isoelectric point (pI) estimated from gel image. ¥ Theoretical MW and pI from Expasy MW/pI calculator (http://www.expasy.ch/tools). € Cathepsin D Active form (aa 65-412). ₡ Cathepsin D Heavy chain (aa169-412). ∆ TPP1 (aa 196-563). ⱡ 3,2-trans-enoyl-CoA isomerase (aa 42-302). ^∗∗^ trend. *δ* without mitochondrial signal sequence.

**Table 2:** Proteins identified from spots changing with AMD (3 or less protein ID spots).

Spot no.†	*p* value	Diff. From MGS 1	Multiple comparisons	Direction/model ‡	Protein ID	Accession UniProt	Gene	Experimental MW/pI §	Theoretical MW/pI ¥	MSMS		
										Sequence coverage	Unique peptides	Total spectra
D1	0.0002	2,3,4	0.0130, 0.0004, 0.0044	I, O	Apolipoprotein A-I	P02647	APOA1	22.8/5.6	28.1/5.27	43	12	23
					GST *π*	P09211	GSTP1		23.3/5.64	35	5	9

D2	0.0185	3 ^∗∗^, 4	0.0614, 0.0294	I, A	Ras-related protein Rab-6A	P20340	RAB6A	22/5.7	23.6/5.54	23	3	3
					GST *π*	P09211	GSTP1		23.3/5.64	26	3	3

D3	0.0068	2,3,4	0.0287, 0.0306, 0.0206	I, O	Superoxide dismutase 1	P00441	SOD1	17.8/6.3	15.8/5.7	29	3	5

D4	<0.0001	Linear		I, L	Peroxiredoxin-3	P30048	PRDX3	22.5/6.7	27.7/7.0	21	3	4

D5	0.0019	2^∗∗^,4^∗∗^	0.0638, 0.0602	I, A	Alpha enolase	P06733	ENO1	47.5/7.1	47.1/7.39	51	16	60
					HMG CoA synthase, mitochondrial	P54868	HMGCS2		52/6.64 ⱡ	18	7	10

D6	0.0014	Linear		I, L	Phosphoglycerate kinase 1	P00558		44.5/7.2	44.6/8.1	30	7	10

D7	0.0168	Linear		I, L	Glutamine synthetase	P15104	GLUL	44.5/7.4	41.9/6.89	26	9	14
					Isocitrate dehydrogenase [NADP] cytoplasmic	O75874	IDH1		46.5/6.53	25	9	14
					Elongation factor Tu, mitochondrial	P49411	TUFM		45.0/6.31 ⱡ	13	4	4

D8	0.0011	2, 3,4	0.0028, 0.0044, 0.0339	I, O	Retinol-binding protein 1	P09455	RBP1	14/5.4	15.8/5.11	54	7	10

D11	0.0171	2	0.0088	D, O	Aldehyde dehydrogenase 9A1	P49189	ALDH9A1	52.8/6.3	53.7/5.69	9	3	3

D12	0.0849^∗∗^	2^∗∗^	0.0655	I, O	Inactive C-alpha-formylglycine-generating enzyme 2	Q8NBJ7	SUMF2	36/6.3	31.2/6.52	20	4	4
					Geranylgeranyl pyrophosphate synthase	O95749	GGPS1		34.9/5.78	13	3	3

D13	0.0048	2,3,4	0.0150, 0.0128, 0.0577	I, O	Inorganic pyrophosphatase 2, mitochondrial	Q9H2U2	PPA2	38.2/6.4	37.9/7.01 ∆	31	7	12
					Retinaldehyde-binding protein 1	P12271	RLBP1		36.3/4.98	19	4	4

D14	0.0061	2,3^∗∗^,4	0.0157, 0.0680, 0.0202	I, O	Peroxiredoxin-3	P30048	PRDX3	23.5/6.8	27.7/7.68	24	4	6

D16	0.0318	Linear		I,L	NADH-ubiquinone oxidoreductase, mitochondrial	P19404	NDUFV2	22.8/6.4	23.8/5.71	44	8	13
					Ras-related protein Rab-7a	P51149	RAB7A		23.4/6.32	29	4	5

D17	0.0009	3	0.0002	I, A	Beta actin	P60709	ACTB	41.3/5.6	41.7/5.29	20	5	9

D18	0.0106	2	0.005	I, O	HSP70, mitochondrial	P38646	HSPA9	66/6.0	68.8/5.44	35	17	35

D19	<0.0001	2,3,4	<0.0001, 0.0018, 0.0016	I, O	Transforming RhoA	P61586	RHOA	24.5/6.3	21.4/5.83	28	4	4

D20	0.0098	2,3	0.0491, 0.0096	I, O	Aldehyde dehydrogenase, mitochondrial	P05091	ALDH2	56.5/6.3	56.4/6.63 ∆	11	4	4

D21	0.0146	2	0.0096	I, O	Protein disulfide-isomerase A3	P30101	PDIA3	59.1/6.3	56.8/5.98 ∆	25	11	20

D22	0.0078	2,3^∗∗^	0.0037, 0.0948	I, O	Enoyl-CoA hydratase, mitochondrial	P30084	ECHS1	29.4/6.6	28.3/5.88	48	14	30
					Phosphoglycerate mutase 1	P18669	PGAM1		28.7/6.75	17	3	3

D23	0.0305	3,4^∗∗^	0.0266, 0.0887	I, A	Aminoacylase 1	Q03154	ACY1	44.5/6.4	45.9/5.77	13	4	4

† Spot number indicated on gel picture in Figure 2 right panel. ≤ 3 proteins identifications are listed in this table. The remaining spots are listed in Table S2. ‡ Direction increasing (I) or decreasing (D); models include onset (O), linear (L), and advanced stage (A). § Experimental molecular weight (MW) of each spot was calculated based on the relative mobility of the MW markers and distance traveled on the 2D gel. Isoelectric point (pI) estimated from gel image. ¥ Theoretical MW and pI from Expasy MW/pI calculator (http://www.expasy.ch/tools). ^∗∗^ trend. € Cathepsin B Active form (aa 80-333). ∆ precursor. ⱡ processed.

## Data Availability

All data generated or analyzed during this study are included in the published article.

## Supplementary Materials

Supplementary Materials All supplementary materials are provided in the supplementary file. Table S1: Donor demographics and clinical information. Table S2: Proteins identified from spots changing with Aging (spots with >3 protein identifications were not included in downstream pathway analysis). Table S3: Proteins identified from spots changing with AMD (spots with >3 protein identifications were not included in downstream pathway analysis). Table S4: Proteins identified from significantly altered spots from our previous studies and included in the IPA analysis.
