# Supplementary material for: Dissecting Regulators of Aging and Age-Related Macular Degeneration in the Retinal Pigment Epithelium
Source: Oxid Med Cell Longev. 2022 Nov 16;2022:6009787. doi: 10.1155/2022/6009787 (PMC9683958; doi:10.1155/2022/6009787)

**Dissecting Regulators of Aging and Age-Related Macular Degeneration in the Retinal Pigment Epithelium**

Pabalu P. Karunadharma ^1,2^, Rebecca J. Kapphahn ^1^, Madilyn Stahl ^1^, Timothy W. Olsen^1,3^, and Deborah A. Ferrington ^1,2^

Supplementary Materials

**Supplemental Table 1. Donor Demographics and Clinical Information***

| **MGS Grade** | **Sample** | **Gender** | | **Age** | | **Cause of Death^€^** |
| --- | --- | --- | --- | --- | --- | --- |
|  | **size (n)** | **M** | **F** | **Mean** | **Range** |  |
|  |  |  |  | **y ^ƍ^ ± SD** |  |  |
| 1 (Young) | 22 | 14 | 8 | 48 ± 9 | 29-60 | cancer (8), liver disease (2), |
|  |  |  |  |  |  | respiratory (4), sepsis (6), PB (1) |
| 1 ^§^ | 28 | 16 | 12 | 74 ± 8 | 61-91 | cancer (11), MS organ failure (1), |
|  |  |  |  |  |  | heart failure (7), respiratory (3), |
|  |  |  |  |  |  | sepsis (4), PB (1), renal disease (1) |
| 2 | 11 | 5 | 6 | 75 ± 12 | 58-93 | cancer (4), heart failure (2), |
|  |  |  |  |  |  | renal disease (1), respiratory (1), |
|  |  |  |  |  |  | sepsis (1), PB (1), SAH (1) |
| 3 | 13 | 7 | 6 | 79 ± 11 | 55-95 | heart failure (8), cancer (2), |
|  |  |  |  |  |  | renal failure (2), respiratory (1) |
| 4 | 12 | 3 | 9 | 83 ± 6 | 74-94 | cancer (2), heart failture (2), |
|  |  |  |  |  |  | renal disease (3), respiratory (3), |
|  |  |  |  |  |  | hemorrhage (1), sepsis (1) |

*Information supplied from Lions Gift of Sight, formerly Minnesota Lions Eye Bank

§ These donors were used as age-matched controls in the AMD comparison and were also included

in the age comparison

^ƍ^ y = Mean age in years for each group

€ Number of donors for each cause of death indicated in parentheses. PB=perforated bowel,

MS organ failure = multiple system organ failure, SAH= subarachnoid hemorrhage

**Supplemental Table 2: Proteins identified from spots changing with Aging (spots with >3 protein identifications were not included in downstream pathway analysis)**

| **Spot No.†** |  |  |  | **Protein ID** | **Accession UniProt** |  | **Experimental MW / pI §** | **Theoretical MW / pI ¥** | **MSMS scaffold** | | |
| --- | --- | --- | --- | --- | --- | --- | --- | --- | --- | --- | --- |
|  | **p value** | **R^2^** | **Direction‡** |  |  | **Gene** |  |  | **Sequence coverage** | **Unique peptides** | **Total spectra** |
| A7 * | 0.0009 | 0.2058 | D | Cytochrome b-c1, mitochondrial | P31930 | UQCRC1 | 45.5 / 6.0 | 52.5 / 5.94 | 51 | 16 | 38 |
|  |  |  |  | Solute carrier family 9 | O14745 | SLC9A3R1 |  | 39 / 5.77 | 21 | 6 | 7 |
|  |  |  |  | Cathepsin D | P07339 | CTSD |  | 44.6 / 6.1 | 16 | 4 | 4 |
|  |  |  |  | Tripeptidyl-peptidase 1 | O14773 | TPP1 |  | 39.8 / 5.75 | 11 | 4 | 7 |
| A8* | 0.0575 ** | 0.0967 | I | Aldehyde dehydrogenase family 9 member A1 | P49189 | ALDH9A1 | 50.5 / 6.1 | 54 / 5.87 | 35 | 16 | 24 |
|  |  |  |  | S-arrestin | P10523 | SAG |  | 45 / 6.57 | 35 | 12 | 15 |
|  |  |  |  | ATP synthase subunit beta, mitochondrial | P06576 | ATP5F1B |  | 57 / 5.40 | 28 | 10 | 11 |
|  |  |  |  | 2-oxoglutarate dehydrogenase complex E2, mitochondrial | P36957 | DLST |  | 49 / 8.95 | 21 | 7 | 16 |
|  |  |  |  | Fibrinogen gamma chain | P02679 | FGG |  | 52 / 5.62 | 10 | 3 | 3 |
|  |  |  |  | Peptidase M20 domain-containing protein 2 | Q8IYS1 | PM20D2 |  | 48 / 5.85 | 12 | 4 | 6 |
|  |  |  |  | Protein disulfide-isomerase A6 | Q15084 | PDIA6 |  | 48 / 5.08 | 13 | 4 | 5 |
|  |  |  |  | Rhodopsin | P08100 | RHO |  | 39 / 6.65 | 11 | 3 | 4 |
|  |  |  |  | RuvB-like 2 | Q9Y230 | RUVBL2 |  | 51 / 5.64 | 7 | 3 | 4 |
|  |  |  |  | Arylsulfatase A | P15289 | ARSA |  | 54 / 6.07 | 6 | 3 | 3 |
| A10* | 0.0256 | 0.1016 | D | Ras-related protein Rab-14 | P61106 | RAB14 | 23.5 / 6.7 | 24 / 5.85 | 71 | 12 | 24 |
|  |  |  |  | Ras-related Rab-11A | P62491 | RAB11A |  | 24 / 6.12 | 34 | 5 | 9 |
|  |  |  |  | Peroxiredoxin-3 | P30048 | PRDX3 |  | 28 / 7.78 | 38 | 7 | 41 |
|  |  |  |  | Transforming protein RhoA | P61586 | RHOA |  | 22 / 6.10 | 24 | 5 | 7 |
| A11* | 0.0051 | 0.1519 | D | ERp29 | P30040 | ERP29 | 26.5 / 6.7 | 29 / 6.77 | 40 | 8 | 19 |
|  |  |  |  | 3,2-trans-enoyl-CoA isomerase | P42126 | ECI1 |  | 28.7 / 6.0 ⱡ | 26 | 6 | 14 |
|  |  |  |  | Phosphoglycerate mutase 1 | P18669 | PGAM1 |  | 28.7 / 6.75 | 30 | 6 | 9 |
|  |  |  |  | Pyridoxine-phosphate oxidase | Q9NVS9 | PNPO |  | 30/7.06 | 16 | 3 | 4 |
|  |  |  |  | Cathepsin D | P07339 | CTSD |  | 26.7 / 5.56 ₡ | 16 | 4 | 4 |
| A13* | 0.001 | 0.3357 | D | HMG CoA Synthase, mitochondrial | P54868 | HMGCS2 | 47.5 / 7.2 | 52 / 8.16 δ | 34 | 14 | 27 |
|  |  |  |  | Alpha enolase | P06733 | ENO1 |  | 47.2 /7.01 | 43 | 12 | 23 |
|  |  |  |  | Rab GDP dissociation inhibitor beta | P50395 | GDI2 |  | 51 / 6.47 | 20 | 6 | 6 |
|  |  |  |  | DnaJ homolog subfamily A2 | O60884 | DNAJA2 |  | 46 / 6.48 | 9 | 4 | 4 |
|  |  |  |  | Sorting nexin-5 | Q9Y5X3 | SNX5 |  | 47/ 6.76 | 9 | 3 | 3 |
|  |  |  |  | Proliferation-associated protein 2G4 | Q9UQ80 | PA2G4 |  | 44 / 6.55 | 18 | 5 | 5 |
|  |  |  |  | Phosphatidylinositol 5-phosphate 4-kinase type-2 gamma | Q8TBX8 | PIP4K2C |  | 47 / 6.84 | 12 | 3 | 3 |
|  |  |  |  |  |  |  |  |  |  |  |  |

| † Spot number indicated on gel picture in Figure 2 left panel | | |  |  |  |  |  |  |  |
| --- | --- | --- | --- | --- | --- | --- | --- | --- | --- |
| ‡ Direction Increasing (I) or Decreasing (D) with age | | |  |  |  |  |  |  |  |
| § Experimental molecular weight (MW) of each spot was calculated based on the relative mobility of the MW markers and distance traveled on the 2-D gel. Isoelectric point (pI) estimated from gel image. | | | | |  |  |  |  |  |
| ¥ Theoretical MW and pI from Expasy MW/pI calculator (www.expasy.ch/tools) | | | |  |  |  |  |  |  |
| € Cathepsin D Active form (aa 65-412) | |  |  |  |  |  |  |  |  |
| ₡ Cathepsin D Heavy chain (aa169-412) | |  |  |  |  |  |  |  |  |
| ∆ TPP1 (aa 196-563) |  |  |  |  |  |  |  |  |  |
| ⱡ 3,2-trans-enoyl-CoA isomerase (aa 42-302) | |  |  |  |  |  |  |  |  |
| ** trend |  |  |  |  |  |  |  |  |  |
| δ without mitochondrial signal sequence | |  |  |  |  |  |  |  |  |

**Supplemental Table 3: Proteins identified from spots changing with AMD (spots with >3 protein identifications were not included in downstream pathway analysis)**

| **Spot No.†** | **p value** | **Diff. from MGS 1** | **Multiple comparisons** | **Direction / model ‡** | **Protein ID** | **Accession UniProt** |  | **Experimental MW/ pI ^§^** | **Theoretical MW/ pI^¥^** | **MSMS** | | |
| --- | --- | --- | --- | --- | --- | --- | --- | --- | --- | --- | --- | --- |
|  |  |  |  |  |  |  | **Gene** |  |  | **Sequence coverage** | **Unique peptides** | **Total spectra** |
| D9* | 0.0380 | 2 | 0.0205 | I, O | Cathepsin B | P07858 | CTSB | 22.8 / 5.4 | 27.8 / 5.22 € | 26 | 8 | 29 |
|  |  |  |  |  | Apolipoprotein A-I | P02647 | APOA1 |  | 28.1 / 5.27 ⱡ | 31 | 7 | 10 |
|  |  |  |  |  | GST π | P09211 | GSTP1 |  | 23.3 / 5.64 | 47 | 7 | 9 |
|  |  |  |  |  | Ubiquitin-conjugating enzyme E2 K | P61086 | UBE2K |  | 22.3 / 5.33 | 28 | 5 | 5 |
|  |  |  |  |  | Ras-related protein Rab-6A | P20340 | RAB6A |  | 23.6 / 5.54 | 29 | 3 | 11 |
|  |  |  |  |  | Ras-related protein Rab-11A | P62491 | RAB11A |  | 23.9 / 6.16 | 20 | 4 | 4 |
|  |  |  |  |  | Catechol O-methyltransferase | P21964 | COMT |  | 27.3 / 5.36 ⱡ | 18 | 3 | 4 |
|  |  |  |  |  | Cathepsin D | P07339 | CTSD |  | 26.7 / 5.56 ₡ | 14 | 3 | 3 |
| D10* | 0.0003 | 2,3,4 | 0.0183, 0.0002, 0.0192 | I, O | Cathepsin D | P07339 | CTSD | 48 / 5.6 | 44.5 /6.1 | 14 | 4 | 4 |
|  |  |  |  |  | Hsc-70 interacting protein | P50502 | ST13 |  | 41.3 / 5.18 | 15 | 4 | 12 |
|  |  |  |  |  | Thioredoxin domain containing-protein 5 | Q8NBS9 | TXNDC5 |  | 47.6 / 5.63 | 9 | 3 | 4 |
|  |  |  |  |  | Protein disulfide-isomerase A6 | Q15084 | PDIA6 |  | 46.2 / 4.95 | 13 | 4 | 4 |
|  |  |  |  |  | Alpha tubulin 1B | P68363 | TUBA1B |  | 50.1 / 4.94 | 10 | 3 | 3 |
|  |  |  |  |  | 26S proteasome regulatory subunit 6A | P17980 | PSMC3 |  | 49.2 / 5.13 | 9 | 3 | 3 |
| D15* | 0.0005 | 2,4 | 0.0013, 0.0113 | D, O | V-type proton ATPase subunit E 1 | P36543 | ATP6V1E1 | 32 / 7.1 | 26.1 / 7.7 | 25 | 5 | 10 |
|  |  |  |  |  | Pyridoxal phosphate phosphatase | Q96GD0 | PDXP |  | 31.7 / 6.11 | 20 | 4 | 5 |
|  |  |  |  |  | Creatine kinase M | P06732 | CKM |  | 27.5 / 9.28 ⱡ | 17 | 4 | 5 |
|  |  |  |  |  | Retinol dehydrogenase 5 | Q92781 | RDH5 |  | 35.0 / 9.47 | 16 | 4 | 4 |
|  |  |  |  |  | Adenylate kinase 1 | P00568 | AK1 |  | 21.6 / 8.73 | 19 | 3 | 3 |
|  |  |  |  |  | Enolase beta | P13929 | ENO3 |  | 46.9 / 7.73 | 11 | 3 | 3 |
|  |  |  |  |  | Retinol dehydrogenase 11 | Q8TC12 | RDH11 |  | 35.4 / 9.05 | 15 | 3 | 3 |

|  |  |  |  |  |  |  |  |  |  |
| --- | --- | --- | --- | --- | --- | --- | --- | --- | --- |

| † Spot number indicated on gel picture in Figure 2 right panel | | | |  |  |  |  |  |  |
| --- | --- | --- | --- | --- | --- | --- | --- | --- | --- |
| ‡ Direction Increasing (I) or Decreasing (D) ; models include onset (O), linear (L), advanced stage (A) | | | | |  |  |  |  |  |
| § Experimental molecular weight (MW) of each spot was calculated based on the relative mobility of the MW markers and distance traveled on the 2-D gel. Isoelectric point (pI) estimated from gel image. | | | | | |  |  |  |  |
| ¥ Theoretical MW and pI from Expasy MW/pI calculator (www.expasy.ch/tools) | | | |  |  |  |  |  |  |
| * spots did not meet criteria of ≤ 3 protein IDs per spot; not included in bioinformatics | | | |  |  |  |  |  |  |
| ** trend |  |  |  |  |  |  |  |  |  |
| ₡ Cathepsin D Heavy chain (aa169-412) | | |  |  |  |  |  |  |  |
| € Cathepsin B Active form (aa 80-333) | | |  |  |  |  |  |  |  |
| ∆ precursor |  |  |  |  |  |  |  |  |  |
| ⱡ processsed |  |  |  |  |  |  |  |  |  |

**Supplemental Table 4: Proteins identified as changing with AMD in other studies**

| **Protein ID** | **Accession UniProt** |  | **Direction / model ‡** | **Source** |
| --- | --- | --- | --- | --- |
|  |  | **Gene** |  |  |
| alpha A crystallin | P02489 | CRYAA | D,O | Nordgaard et al, 2006; Table 2 |
| ATP synthase subunit alpha, mitochondrial | P25705 | ATP5F1A | D,L | Nordgaard et al, 2008 |
| ATP synthase beta, mitochondrial | P06576 | ATP5F1B | D,O | Nordgaard et al, 2006; Table 2 / Nordgaard et al, 2008; Fig. 4 |
| ATP synthase subunit delta, mitochondrial | P30049 | ATP5F1D | D,A | Nordgaard et al, 2008 |
| Catalase | P04040 | CAT | I, L | Decanini et al, 2007 |
| COX VIb | P14854 | COX6B1 | D,A | Nordgaard et al, 2008 |
| CRABP 1 | P29762 | CRABP1 | D,L | Nordgaard et al, 2006; Table 2 |
| CRALBP | P12271 | RLBP1 | D,O | Nordgaard et al, 2006; Table 2 |
| eIF-4H | Q15056 | EIF4H | I,A | Nordgaard et al, 2006; Table 2 |
| Elongation factor Tu, mitochondrial | P49411 | TUFM | I,L | Nordgaard et al, 2008 |
| GST pi | P09211 | GSTP1 | D,L | Nordgaard et al, 2006; Table 2 |
| Heat shock 70 kDa protein 1A (HSP70) | P0DMV8 | HSPA1A | D,O | Nordgaard et al, 2006; Table 2 |
| Heat shock cognate 71 kDa protein (HSC 70) | P11142 | HSPA8 | D,O | Nordgaard et al, 2006; Table 2 |
| Heat shock protein 60 kDa, mitochondrial (HSP60) | P10809 | HSPD1 | D,O | Nordgaard et al, 2006; Table 2 |
| HSP27 | P04792 | HSPB1 | I, L | Decanini et al, 2007 |
| HSP90 alpha | P07900 | HSP90AA1 | I, L | Decanini et al, 2007 |
| HSP90 beta | P08238 | HSP90AB1 | I, L | Decanini et al, 2007 |
| Insulin receptor | P06213 | INSR | I, L | Decanini et al, 2007 |
| Mitofilin | Q16891 | IMMT | I,L | Nordgaard et al, 2008 |
| mt HSP ; 70Stress-70 protein, mitochondrial (GRP75) | P38646 | HSPA9 | D,A | Nordgaard et al, 2006; Table 2 / Nordgaard et al, 2008; Fig. 4 |
| Proteasome 20S Alpha 6 | P25786 | PSMA1 | I, L | Decanini et al, 2007 |
| Proteasome 20S Alpha 7 | P25788 | PSMA3 | I, L | Decanini et al, 2007 |
| Pyruvate kinase PKM | P14618 | PKM | I,L | Nordgaard et al, 2006; Table 2 |
| Superoxide dismutase [Mn], mitochondrial (Mn SOD) | P04179 | SOD2 | I, L | Decanini et al, 2007 |
| VDAC-1 | P21796 | VDAC 1 | I,L | Nordgaard et al, 2006; Table 2 |

**Supplementary Figure 1:**

1. **Aging Upstream Regulators.**

**
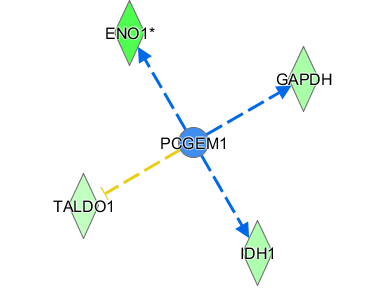
**

**
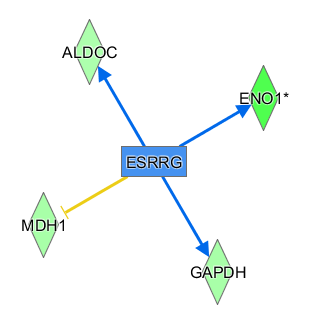
**
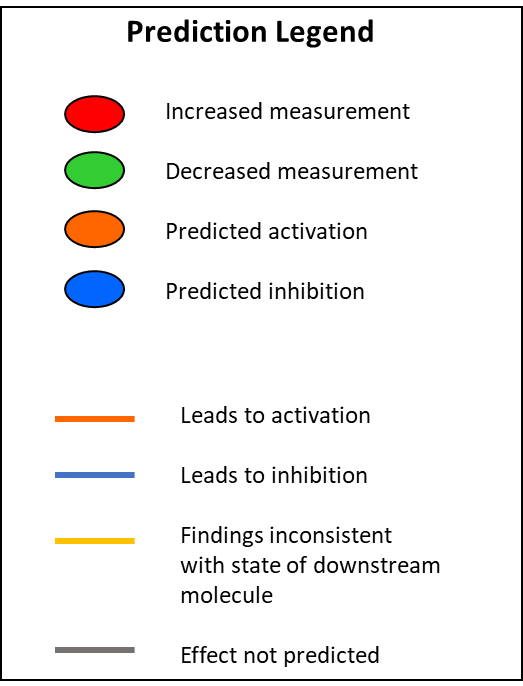


Ingenuity (IPA, Qiagen Inc.) upstream regulator analysis identified 5 regulator molecules significantly affected with Aging. Prostate-specific transcript 1 (PCGEM1) and Estrogen related receptor gamma (ESRRG) with affected targets are shown here. Other regulators are shown in Fig. 6A.

**Supplementary Figure 1:**

1. **AMD Upstream Regulators.**


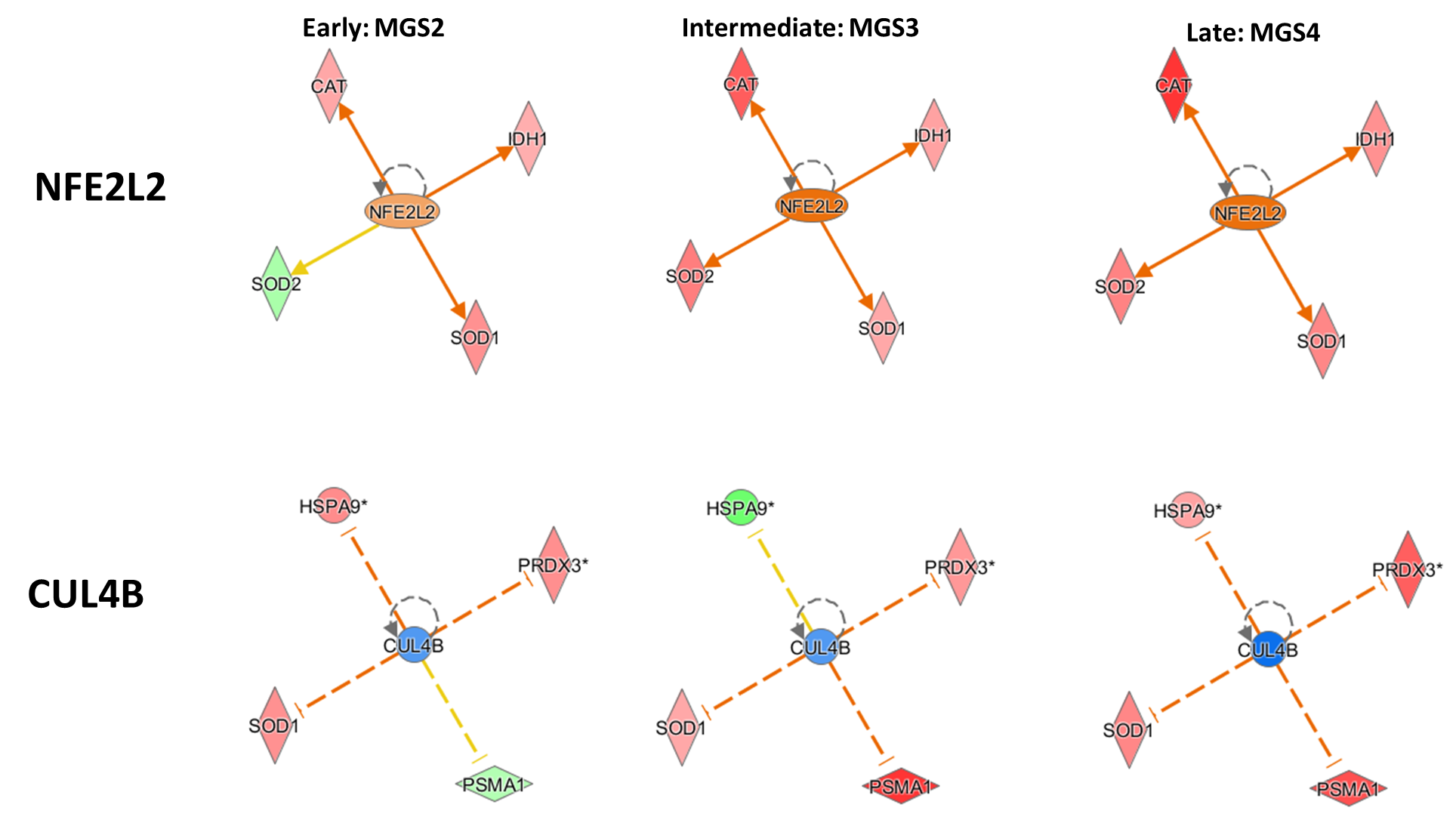


Ingenuity (IPA, Qiagen Inc.) upstream regulator analysis identified 12 regulator molecules predicted to be significantly affected with AMD. HSF1, TCR, and LonP1 with interacting proteins are shown in Figure 6B. NFE2L2, CUL4B, INFG, IL15, MYC, TNF, HIF1 alpha, PCGEM1, and TRAP1 are shown in this figure.

**Supplementary Figure 1:**

**(B) AMD Upstream Regulators.**


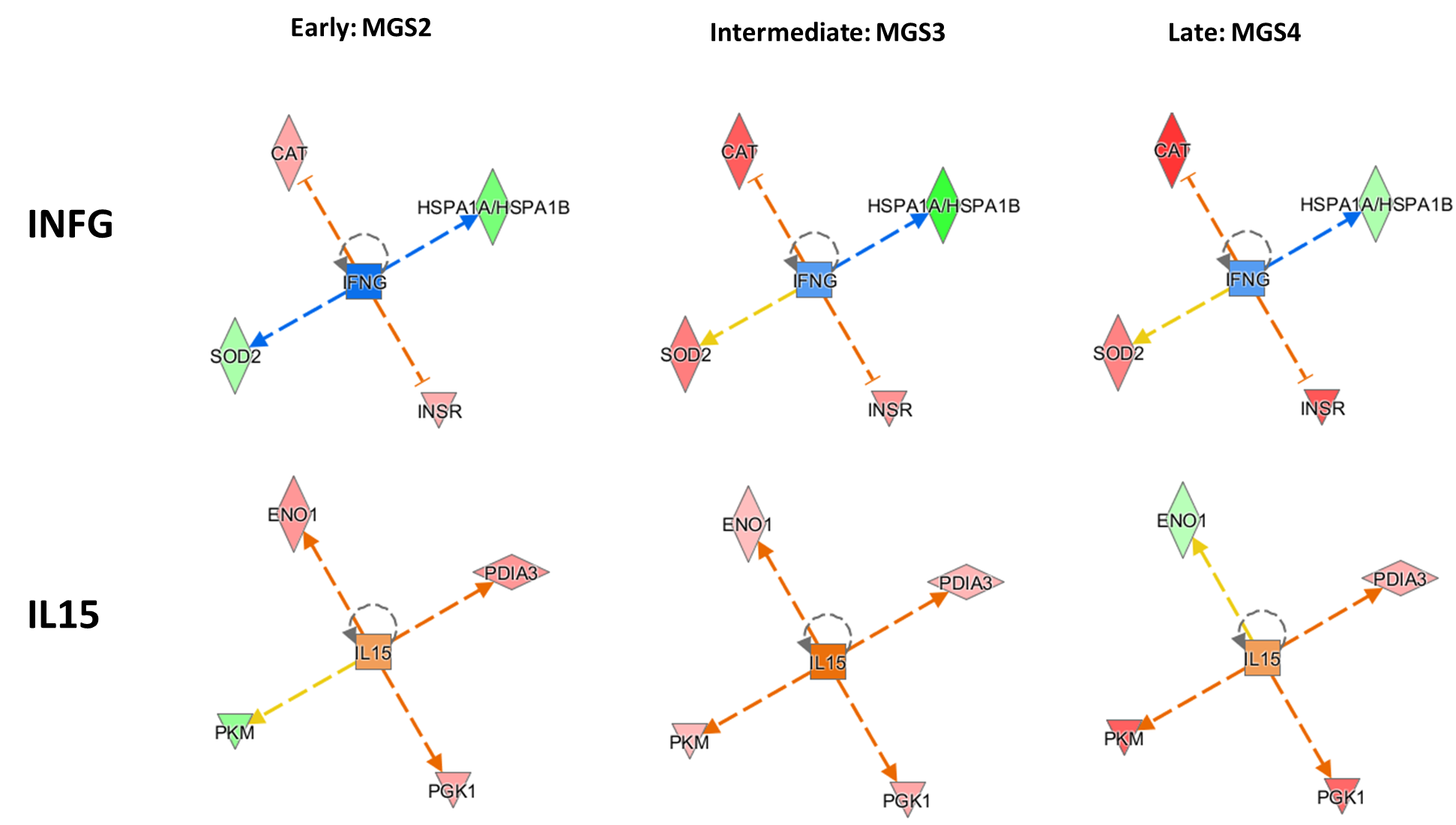


**Supplementary Figure 1:**

1. **AMD Upstream Regulators.**


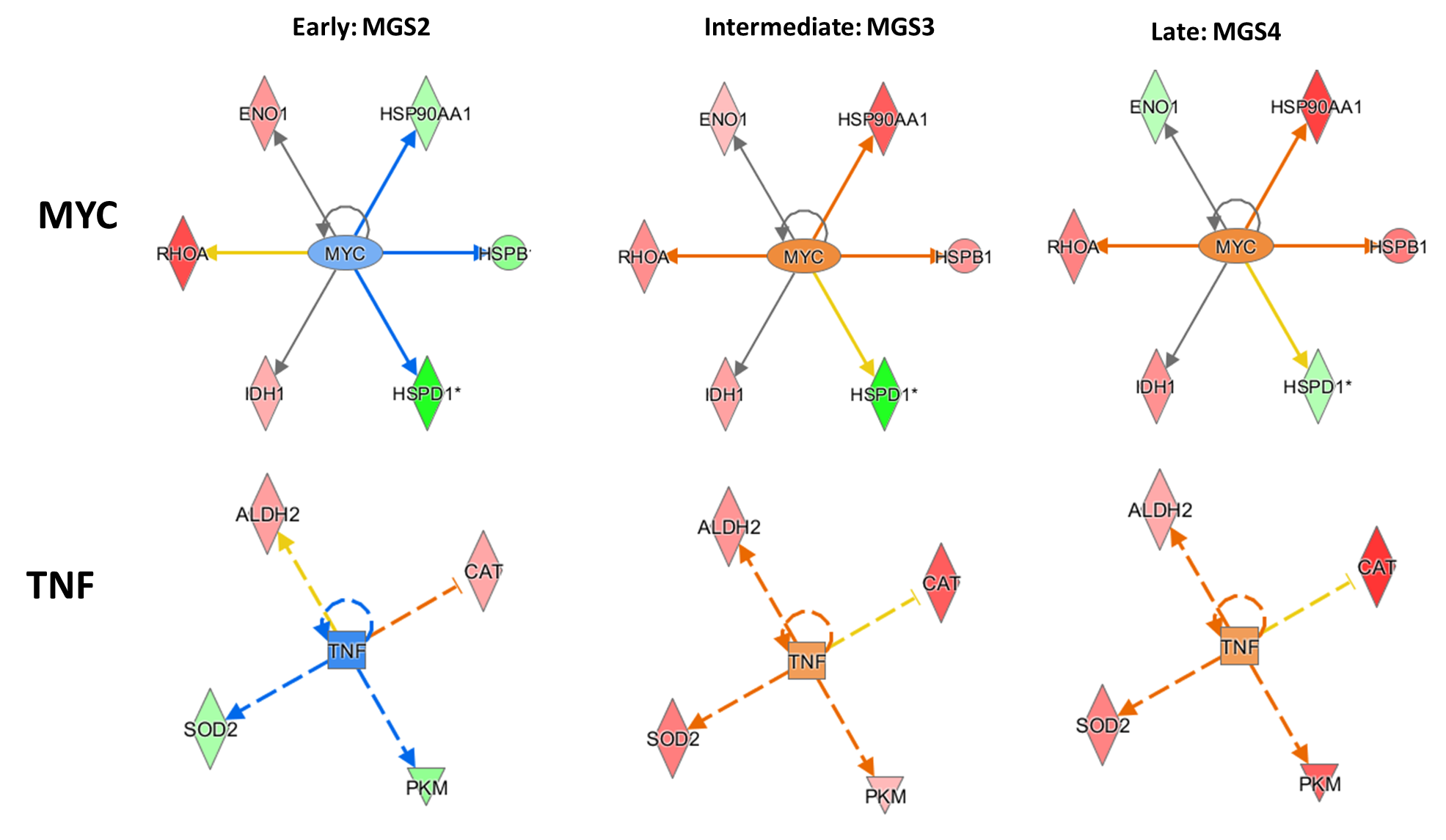


**Supplementary Figure 1:**

1. **AMD Upstream Regulators.**


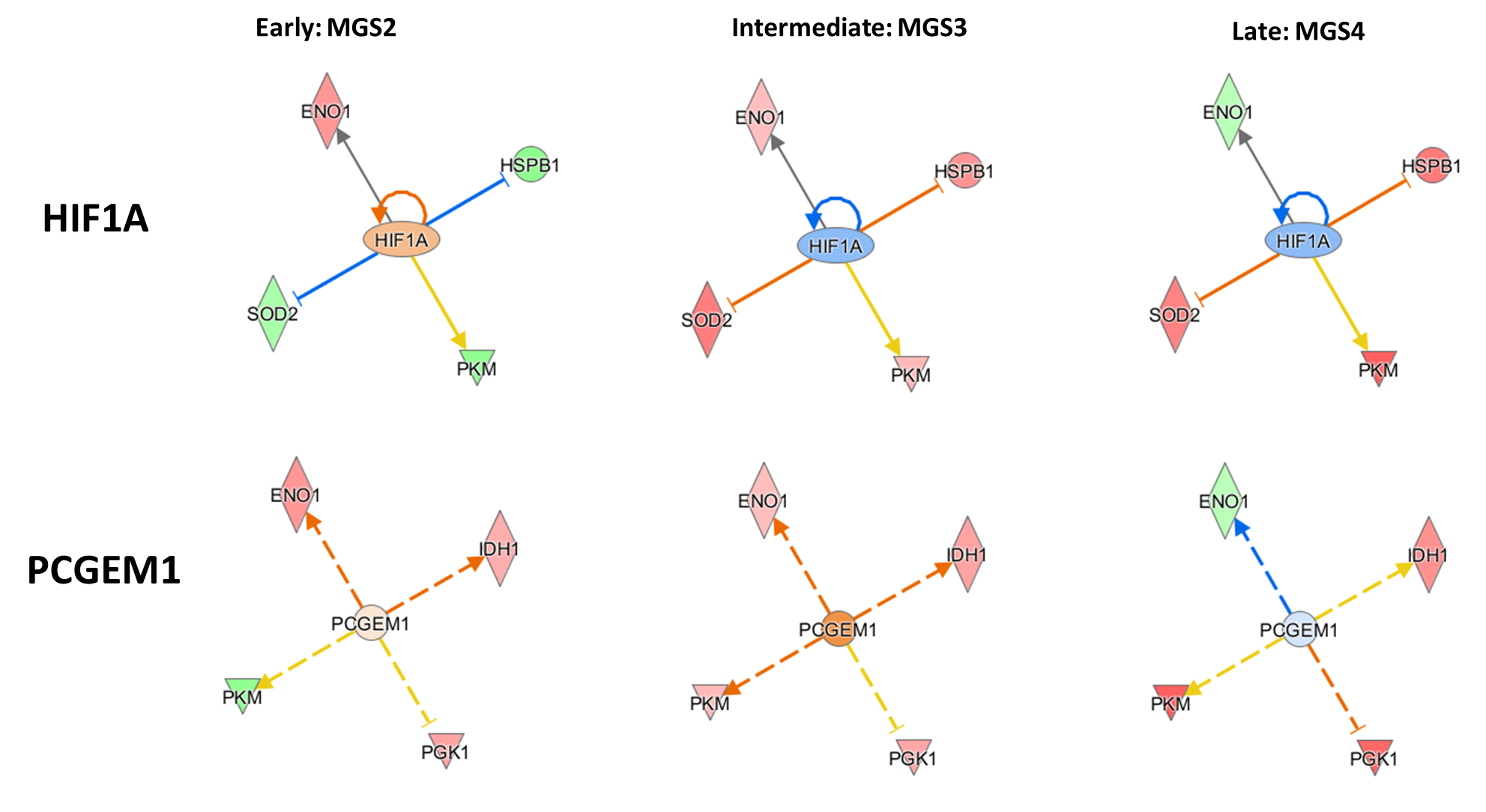


**Supplementary Figure 1:**

1. **AMD Upstream Regulators.**


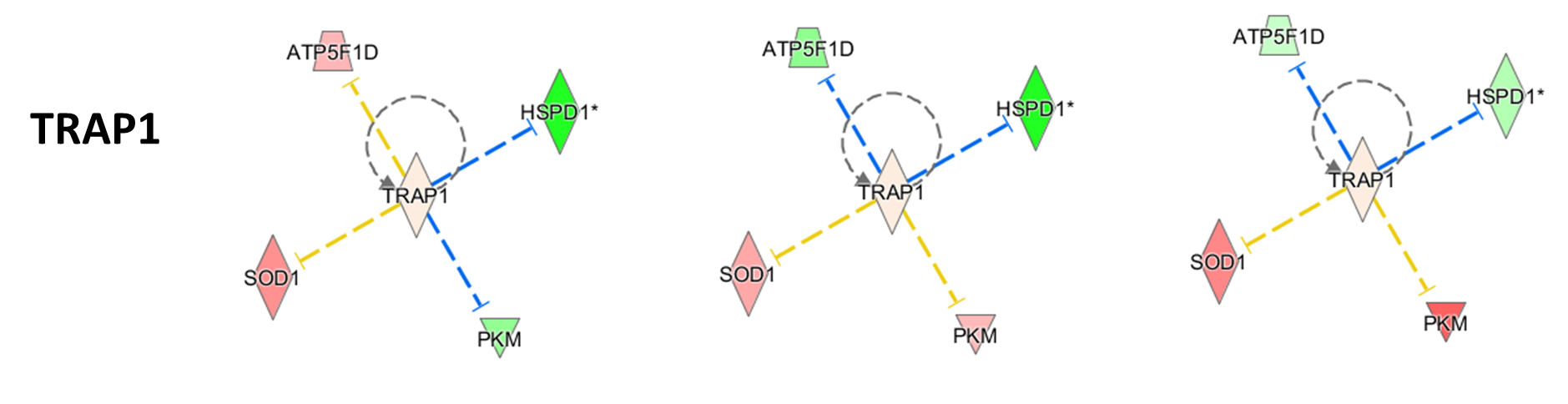

Supplement: Supplementary Materials — All supplementary materials are provided in the supplementary file. Table S1: Donor demographics and clinical information. Table S2: Proteins identified from spots changing with Aging (spots with >3 protein identifications were not included in downstream pathway analysis). Table S3: Proteins identified from spots changing with AMD (spots with >3 protein identifications were not included in downstream pathway analysis). Table S4: Proteins identified from significantly altered spots from our previous studies and included in the IPA analysis. [file 6009787.f1.docx]
